# *In situ* characterisation of MnS precipitation in high carbon steel

**DOI:** 10.1038/s41598-019-46450-y

**Published:** 2019-07-12

**Authors:** Yasuhiro Tanaka, Farshid Pahlevani, Suk-Chun Moon, Rian Dippenaar, Veena Sahajwalla

**Affiliations:** 1 Centre for Sustainable Materials Research and Technology (SMaRT), School of Materials Science and Engineering, UNSW Sydney, Kensington, New South Wales 2052 Australia; 2 Steelmaking Division, Yawata works, Nippon Steel Corporation, 1-1 Tobihatacho, Tobata-ku, Kitakyushu, Fukuoka 804-8501 Japan; 3 School of Mechanical, Materials, Mechatronic and Biomedical Engineering, Faculty of Engineering & Information Science, University of Wollongong, Wollongong, New South Wales 2522 Australia

**Keywords:** Metals and alloys, Imaging techniques

## Abstract

Manganese sulphide (MnS) is one of the major non-metallic inclusions in steel with huge impact on steel property. In the case of high carbon steel, due to higher sulphur content and its brittleness, controlling MnS formation is one of the main issues. MnS has a complicated precipitation mechanism during solidification in liquid and solid steel and at the interface with oxide inclusions. Higher sulphur content, lower melting point and different oxide inclusions in high carbon steel will cause MnS precipitation at different stages. In this study, different stages of MnS precipitation from liquid and/or solid in high carbon steel and at the interface with oxide inclusion were investigated comprehensively via two different types of High Temperature Confocal Scanning Laser Microscope (HTCSLM). Samples were analysed further using SEM-EDS for better understanding the pertaining mechanisms. MnS precipitation on the surface of liquid steel was observed *in situ* in a HTCSLM by the use of a concentric solidification technique. Additionally, formation of MnS following solidification and at the interfaces of oxide inclusions, was investigated *in situ* in a HTCSLM, which has a uniform temperature profile across the specimen. These comprehensive descriptions about different stages of MnS precipitation in high carbon steel have been conducted for the first time and provide crucial information for controlling MnS morphology in high carbon steel.

## Introduction

Controlling inclusions during the refining processes of steel is an essential step for producing high quality steel. Many studies have been conducted in this area of inclusion engineering, including issues specifically relating to high carbon steel. It is important to understand how non-metallic inclusions form during solidification of liquid steel, and manganese sulphide (MnS) is arguably the most important non-metallic inclusion in this category. During cooling, sulphur solubility in steel is considerably reduced and sulphide phases will form^1,2^. Ferrous sulphide (FeS) will be the first sulphide phase to form, predominantly at grain boundaries, leading to hot-shortness. In order to guard against hot-shortness, the precipitation of FeS is suppressed by the addition of manganese to the steel so that MnS forms in preference to FeS^1^. MnS particles also enhance machinability in free cutting steels, and it can be used to control grain size in oriental electromagnetic steel material^3^. Therefore, controlling the formation of MnS is very important in achieving the required performance of different steel products.

Previous studies indicated that MnS can precipitate in liquid low carbon steel, but it can also crystallise in solid steel. Takada *et al*.^4^ have shown that the morphology of MnS depends on the sulphur content in the base steel, and moreover, that a huge MnS dendrites can precipitate from liquid steel when the sulphur content is so high that the composition falls within the co-existence area of MnS and liquid Fe as shown in the ternary Fe-Mn-S phase diagram. On the other hand, Hasegawa *et al*.^5^ and Ueshima *et al*.^6^ investigated MnS nucleation and growth in solid δ-ferrite and γ-austenite in an Fe-Si alloy. Sims and Dahle^7^ categorized MnS morphologies into three groups; globular, or angular phases, which precipitated from liquid steel, and a rod-like phase, which crystallises in solid steel. Oikawa *et al*.^8^ revealed that morphologies of primary MnS, which is formed from the liquid, and secondary MnS, formed in the solid, are highly dependent on carbon and silicon content of the steel. Schwerdtfeger^9,10^ derived an empirical equation, describing the relation between the mean radius of the primary MnS phase and cooling rate in a 0.55%C steel. Diederichs *et al*.^11,12^ modified the model developed by Schwerdtfeger^9,10^ by considering the concentrations of manganese and sulphur, as well as the dendrite arm spacing in medium-carbon steel.

Another important type of MnS forms at the interface with oxide inclusions. Yuki *et al*.^13^ investigated *in situ* MnS formation behaviour on a Fe-Ni alloy surface by using Confocal Scanning Laser Microscope (CSLM) and concluded that MnS phases are formed at the surface of Al_2_O_3_ particles. Wakoh *et al*.^14^ also investigated MnS nucleation at the surface of various oxide particles and indicated that oxide particles are likely to act as MnS nucleation sites when the sulphur content is higher than 0.01% in a 1% Mn steel. It was suggested that Mn-Ti oxide inclusions most likely act as nucleation sites^14^. Ueshima *et al*.^15^ contended that MnS can nucleate at the surface of a variety of oxide particles and that the Mn-depleted zone around such particles is likely to be a nucleation site of α-ferrite.

Many previous studies have focused on low or medium carbon steel. However, high carbon steel received much less attention notwithstanding the fact that they attracted much attention in recent years due to their superior mechanical properties and the potential of applications in harsh environments. These high-carbon steels contain high sulphur and manganese and MnS plays a significant in the determination of the properties of these steels. Moreover, high carbon steels are mainly deoxidized by silicon, leading to the formation of non-alumina based, semi-liquid oxide inclusions, which are very different than the inclusions formed in Al-killed low carbon steel. Hence, comprehensive understanding of the different stages of MnS precipitation in high carbon steel is crucial. The aim of the present study is to conduct a comprehensive study of the different stages of MnS precipitation/crystallisation from liquid/solid high carbon steel and at the interface with oxide particles. Consequently, the outcomes of this study are likely to provide a valuable contribution to the production of high-performance high carbon steel.

## Experimental Procedure

### Experimental design to assess the formation of different types of MnS

**Figure 1:**
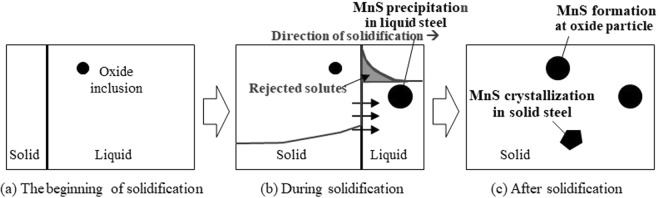
Schematic illustration of three types of MnS phase and different stages of precipitation.

Figure 1 illustrates a schematic diagram of three types of MnS and their formation stages in high carbon steel: precipitation in liquid steel; crystallisation in solid steel; and formation at oxide particle interface. Regarding the precipitation in liquid steel, the solubility product of MnS, which means the soluble limit of manganese and sulphur and is expressed with [%Mn] multiplied by [%S], is required to be higher than the critical value. For example, Oikawa *et al*.^16^ derived an equation below about the critical value of low carbon steel based on the study of Sano *et al*.^17^;

1 $$\mathrm{log}([ \% Mn]\times [ \% S])=-\frac{9200}{T}+5.3$$

where *T* is the temperature (K). If the solidification of steel samples can be controlled in a way that micro segregation during solidification is enhanced, then [%Mn] and [%S] in liquid steel can be controlled. For the observation of MnS formation from the liquid where some amount of enrichment with solutes exists by segregation, it is necessary to utilize a furnace in which the steel sample has a controlled temperature gradient which will enable the solidification with segregation. On the other hand, in order to observe the MnS crystallisation in solid steel, it is important to keep the composition uniform throughout the sample, still requiring melting sample for dissolving all pre-existing precipitates prior to MnS crystallization. In this case, it is appropriate to use a furnace which has a uniform temperature profile across the sample. In this type of furnace, less segregation occurs during solidification due to random nucleation at multiple sites because of the uniform temperature across the specimen. Therefore, the composition and temperature of the base steel become almost uniform throughout the sample after solidification. For the same reason, MnS formation at the interface with oxide particle need to be investigated in the furnace with a uniform temperature profile. Hence, two types of furnaces were used for the *in-situ* observation.

### Sample dimensions and basic procedure of CSLM

**Figure 2:**
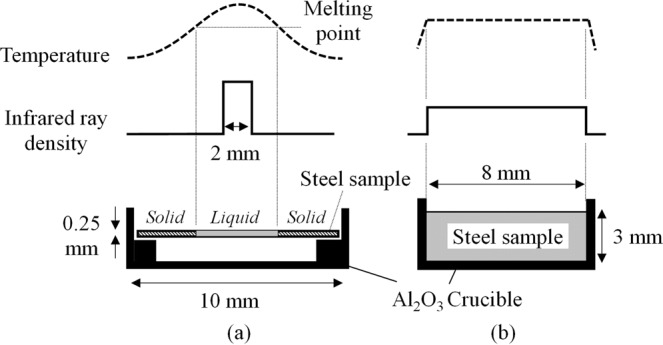
Schematic illustration of the sample dimensions, infrared ray densities and temperature profiles in a sample in the two different types of infrared ray furnace in HTCSLM. ((**a**) Type-1 furnace, (**b**) Type-2 furnace).

The sample dimensions, infrared ray density and temperature profile of the two types of furnace are schematically illustrated in Fig. 2. In the Type-1 furnace, shown in Fig. 2(a), infrared ray focuses on the centre part of the sample within a 2 mm diameter. Therefore, a temperature gradient from the centre where the heat is concentrated directly towards the outer part of the sample where the heat is conducted into the solid rim. This feature enables us to observe a circular liquid pool when the temperature at the centre of the specimen is kept above the liquidus temperature. In addition, given appropriate cooling conditions, a stable planar solid/liquid solidification front can be induced and observed *in situ* by the so-called a concentric solidification technique, developed by Reid *et al*.^18^. In Type 2 furnace, the temperature is evenly distributed on the surface of the specimen as shown in Fig. 2(b), which leads to a uniform temperature profile ensuring a uniform composition after solidification.

In the Type-2 furnace, the control thermocouples are positioned beneath the crucible within a uniform temperature zone. As a result, the measured temperature represents the temperature of sample. In the Type-1 furnace, the control thermocouples are located in the platinum holder outside the crucible and a calibration is required to determine the actual specimen temperature. For this purpose, the liquidus temperature of each sample was calculated from its chemical composition. During the heating process, high purity argon gas (O_2_ <2 ppm, H_2_O <3 ppm) was used as the atmosphere in both furnaces to avoid oxidation. In addition, the furnace chamber was initially purged, and the argon gas passed through a gas train to remove the remaining oxygen and moisture.

### High carbon steel specimens and heating/cooling pattern

**Figure 3:**
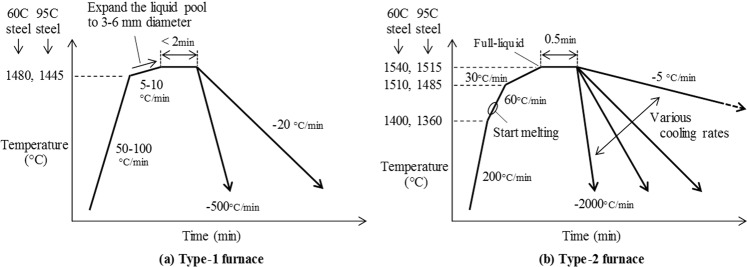
Schematic patterns of heating and cooling treatment with 60 C and 95 C steel. ((**a**) Type-1 furnace, (**b**) Type-2 furnace).

Table 1 shows the chemical compositions, liquidus and solidus temperature of two types of high carbon steel sample used in this study. The liquidus and solidus temperatures for these two different steels were calculated by Eq. 2 derived by Miettinen *et al*.^19^ and Eq. 3 derived by Gryc *et al*.^20^, respectively.

2 $$\begin{array}{rcl}{T}_{Liq} & = & 1538-76.24{C}_{C}-10.35{({C}_{C})}^{2}-11.66{C}_{Si}-4.35{C}_{C}{C}_{Si}-5.62{C}_{Mn}\\  &  & -0.22{C}_{C}{C}_{Mn}-1.95{C}_{Cr}-0.03{C}_{C}{C}_{Cr}-2.2{C}_{Mo}-0.85{C}_{C}{C}_{Mo}-3.58{C}_{Ni}\\  &  & -0.84{C}_{C}{C}_{Ni}-24.78{C}_{P}-12.94{C}_{C}{C}_{P}-32.81{C}_{S}-17.72{C}_{C}\end{array}$$

3 $$\begin{array}{rcl}{T}_{Sol} & = & 1536-251{C}_{C}-12.3{C}_{Si}-6.8{C}_{Mn}-123.4{C}_{P}\\  &  & -\,183.9{C}_{S}-3.3{C}_{Ni}-1.4{C}_{Cr}-3.1{C}_{Cu}-3.6{C}_{Al}\end{array}$$

where *T*_*Liq*_ and *T*_*sol*_ are the liquidus and solidus temperature (°C) respectively. *C*_*X*_ is the weight percentage of element X (wt%). The heating patterns for each sample in Type-1 and Type-2 furnace are demonstrated in Fig. 3. The reduction in heating rate close to the melting point is designed to avoid overheating. In Iype-1 furnace, the liquid pool is expanded by heating with a low heating rate between 5–10 °C/min until it reaches the desired liquid pool size. After holding at temperature for 2 minutes in order to stabilize the liquid pool, the sample is cooled at rates of −20 and −500 °C/min respectively in Type-1 furnace and between −5 to −2000 °C/min in Type-2 furnace.

**Table 1:** Chemical composition and liquidus, solidus temperature of two types of used high carbon steel in this study.

Steel	C	Si	Mn	P	S	Cr	Mo	Ni	T. Al	T. Ca	T. O	*T*_*liq*_	*T*_*sol*_
	(wt %)	(wt %)	(wt %)	(wt %)	(wt %)	(wt %)	(wt %)	(wt %)	(wt %)	(ppm)	(ppm)	(°C)	(°C)
60 C	0.58	0.26	0.74	0.014	0.022	0.20	0.02	0.07	0.004	8.8	30.9	1480	1376
95 C	0.95	0.23	0.88	0.012	0.028	0.23	0.12	0.06	0.002	7.9	16.0	1445	1282

During cooling, the images as well as the measured temperature and time are recorded in video format at a rate of 30 frames per second.

After completion of an experiment, MnS phases on the surface and cross section of each sample were determined in a SEM-EDS microscope (Hitachi S-3400N SEM, Bruker XFlash SDD-EDS) at a beam energy of 10 kV.

## Results and Discussion

### Type A manganese sulphide precipitation in liquid steel

**Figure 4:**
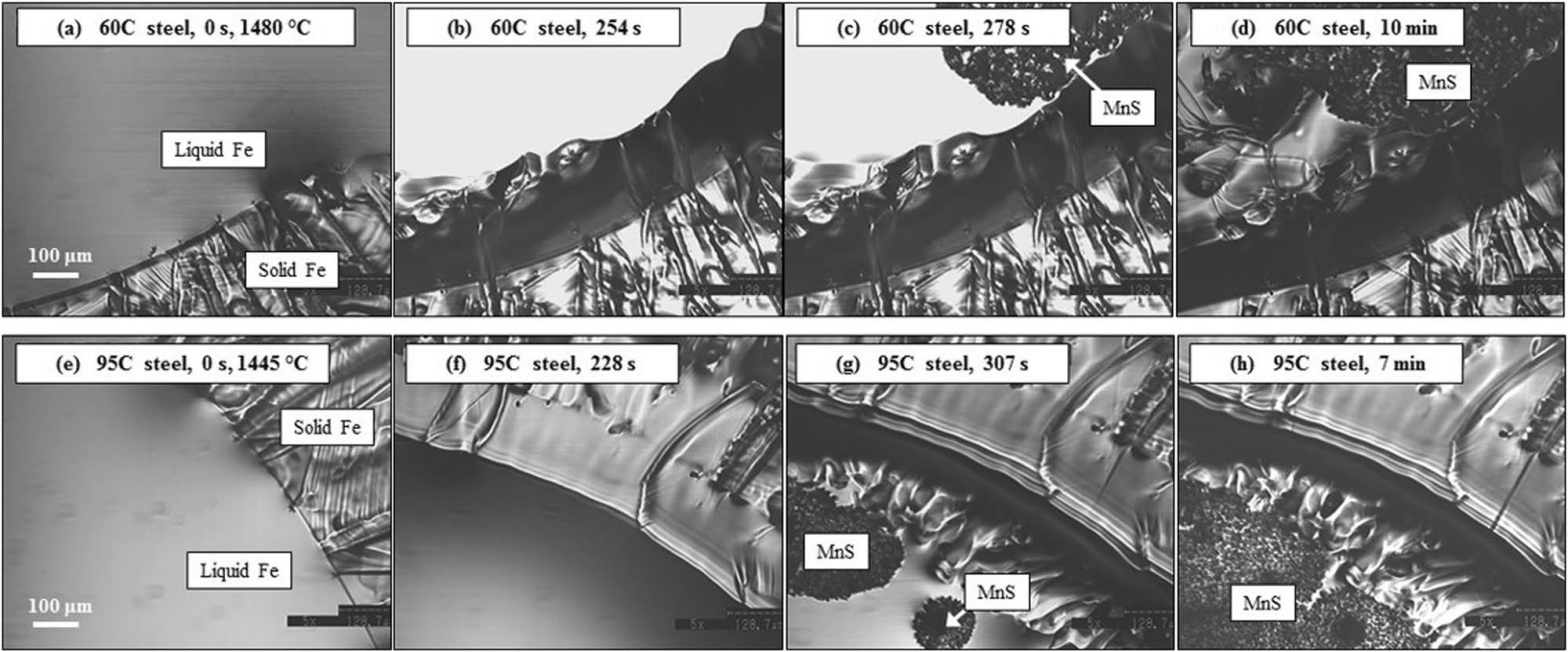
*In situ* observation of solidification and MnS formation on liquid 60 C steel (**a**–**d**) and 95 C steel (**e**–**h**) in type-1 furnace.

**Figure 5:**
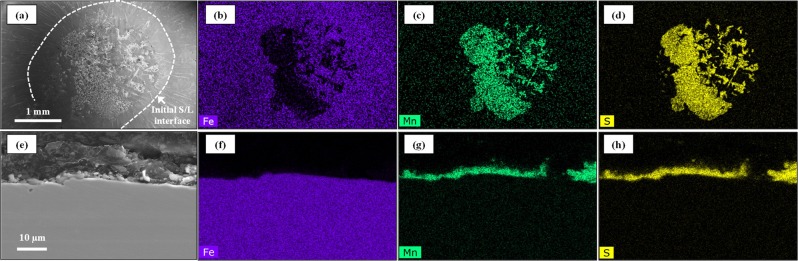
SEM (SE) images and EDS mapping results of MnS phase formed in liquid 60 C steel cooled with −20 °C/min. (**a**–**d**) Surface, (**e**–**h**) Cross section, (**a**,**e**) SEM image, (**b**,**f**) EDS mapping of Fe, (**c**,**g**) EDS mapping of Mn, (**d**,**h**) EDS mapping of S.

**Figure 6:**
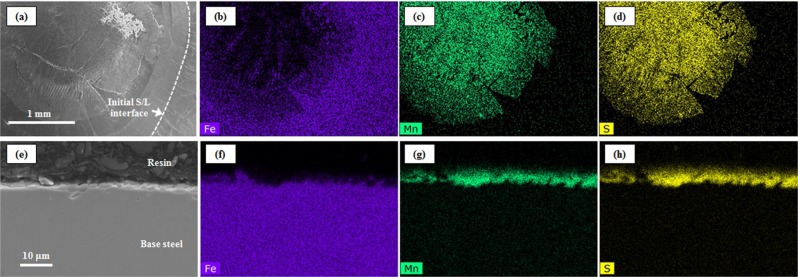
SEM (SE) images and EDS mapping results of MnS phase formed in liquid 95 C steel cooled with −20 °C/min. ((**a**–**d**) Surface, (**e**–**h**) Cross section, (**a**,**e**) SEM image, (**b**,**f**) EDS mapping of Fe, (**c**,**g**) EDS mapping of Mn, (**d**,**h**) EDS mapping of S.

Figure 4 presents the *in-situ* observation of MnS formation in steel types 60 C (a-d) and 95 C (e-h) respectively in the Type-1 furnace by the concentric solidification technique. On the initiation of solidification, shown in Fig. 4(a,e), the temperature at the solid/liquid interface is that of the liquidus, 1480 °C for steel 60 C (image (a) and 1445 °C for steel 95 C (image (e), respectively. The cooling rate in each was −20 °C/min. Following progression of the solidification front towards the centre of the sample, the liquid/solid interface is stable and planar as presented in Fig. 4(b,d). Eventually, MnS phase starts to precipitate in the liquid phase ahead of the solid/liquid interface as shown in Fig. 4(c,g). At this stage, the solidification front becomes unstable and independent nucleation sites are observed ahead of the solid/liquid interface. Finally, solidification is completed as shown in Fig. 4(d,h). Figures 5 and 6 present secondary electron (SE) images in the SEM in addition to EDS mapping results of the MnS phase in the steels 60 C and 95 C respectively. In Figs 5(a) and 6(a), the initial solid/liquid interfaces, which are the same as those shown in Fig. 4(a,e), are highlighted with a white dotted line. It follows from Figs 5(b–d) and 6(b–d), that the phase shown a MnS in Fig. 4 and delineated by the white dotted line in Figs 5 and 6 is indeed MnS. The observation of the cross sections, shown in Figs 5(e–h) and 6(e–h), clearly shows that MnS forms at the solid/liquid interface, which is to be expected since sulphur is a surface active element.

The areas (mm^2^) of the initial liquid pool and MnS precipitation have been measured by analysing the derived SEM-EDS images. Table 2 presents each area (mm^2^) and the ratio of MnS precipitation area to the initial liquid pool area, *R*_*MnS*_, of 60 C and 95 C steel cooled with −20 and −500 °C/min. As shown in this table, the area ratio *R*_*MnS*_ of higher cooling rate is bigger than that of lower cooling rate. The reason is considered to be due to the difference of micro segregation behaviour and severe build-up of carbon, sulphur and manganese in the liquid pool, which will influence the chemical potential of manganese and sulphur^16^.

**Table 2:** The areas of the liquid pool (including subsequent MnS phase) and MnS phase and the ratio of the areas in 60 C steel.

Steel	Cooling	Area		
	rate	Initial	MnS	The area ratio
		liquid pool	precipitation	*R*_*MnS*_
	(°C)	(mm^2^)	(mm^2^)	(%)
60 C	−20	6.9	2.1	31%
	−500	10.5	3.8	36%
95 C	−20	10.6	6.0	57%
	−500	13.5	9.4	70%

MnS phase will precipitate in liquid steel when the solubility product of MnS exceeds the critical value. Oikawa *et al*.^16^ revealed the relationship between the solubility product of MnS and solubility in low carbon steel only, shown in Eq. 1, and it is therefore necessary to consider the thermodynamic interactions with carbon in the high carbon steels of this investigation. In the present study, the theoretical solubility product of MnS was estimated from the following equations;

4 $${\rm{\Delta }}{G}_{MnS}^{0}=-39469+21.71T=-RTln(\frac{1}{{a}_{Mn}{a}_{S}})$$

5 $${a}_{Mn}={f}_{Mn}[ \% Mn]$$

6 $$\mathrm{log}\,{f}_{Mn}\approx {e}_{Mn}^{C}[ \% C]+{e}_{Mn}^{Si}[ \% Si]+{e}_{Mn}^{Mn}[ \% Mn]$$

7 $${a}_{S}={f}_{S}[ \% S]$$

8 $$\mathrm{log}\,{f}_{S}\approx {e}_{S}^{C}[ \% C]+{e}_{S}^{Si}[ \% Si]+{e}_{S}^{Mn}[ \% Mn]$$

where ${\rm{\Delta }}{G}_{MnS}^{0}$ is the standard Gibbs free energy (cal/mol) for the reaction Mn(%) + S(%) = MnS occurring in liquid steel, *R* is the gas constant (cal/K/mol), *T* is the temperature (K) and *a*_*X*_ is the activity (%) of X. Equation 4 was derived by Ueshima *et al*.^21^. Table 3 summarizes the interaction coefficients ${{\boldsymbol{e}}}_{{\boldsymbol{i}}}^{{\boldsymbol{j}}}$ with respect to Eqs 6 and 8, derived by Tanahashi *et al*.^22^ and Sanbongi and Ohtani^23^.

**Table 3:** Interaction coefficient ${{\boldsymbol{e}}}_{{\boldsymbol{i}}}^{{\boldsymbol{j}}}$.

		*j*			Reference
		C	Si	Mn	
*i*	Mn	−0.041	0.103	0	22
	S	0.120	0.065	−0.025	23

${\rm{\Delta }}{G}_{MnS}^{0}$ in Eq. 4 and the interaction coefficients in Table 3 have been validated for low carbon steels only. The liquidus and solidus temperature of high carbon steel are lower than those of low carbon steel and moreover, the pertaining thermodynamic data for the high carbon steels are not available in published literature. In this study, these thermodynamic values were estimated by extrapolation of Eq. 4 and are shown in Table 3. The solubility product of MnS in the liquid steel can be calculated by multiplying [%Mn] in Eq. 5 by [%S] in Eq. 7, and the results are summarized in Table 4.

**Table 4:** Comparison between [%Mn] × [%S] when MnS start precipitating and the solubility product of MnS.

	[%Mn]×[%S] in initial liquid pool			[%Mn]×[%S] when MnS precipitates		Solubility product of MnS
	[%Mn]_ini_	[%S]_ini_	[%Mn]_ini_ × [%S]_ini_	Area ratio *R*_*MnS*_	[%Mn]_pre_ ×[%S]_pre_	
60 C steel	0.74	0.022	0.016	37%	0.12	0.30
95 C steel	0.88	0.028	0.025	70%	0.05	0.21

*In the case of the fastest cooling rate, −500 °C/min.

In order to compare the theoretical solubility product to the estimated values, some parameters are defined as below;

9 $${[ \% X]}_{pre}={[ \% X]}_{ini}\times {R}_{MnS}$$

where [%X]_pre_ is the estimated percentage of X in the liquid steel when MnS phase starts precipitating on the liquid steel surface during solidification; [%X]_ini_ is the percentage of X in the liquid steel before solidification starts. This estimation is based on the rough assumption that all the manganese and sulphur are pushed into the remaining liquid pool as the solid/liquid interface progresses in the course of solidification plane and hence, the liquid is enriched in solute concentrations. Consider the highest cooling rate, −500 °C/min in this study: The product [%Mn]_pre_ × [%S]_pre_ is proportional to *R*_*MnS*_ squared because each is in proportion to *R*_*MnS*_. Table 4 shows that the estimated values of [%Mn]_pre_ × [%S]_pre_ are lower than the theoretical solubility product of MnS in both 60 C and 95 C steels. The most probable reason for this discrepancy is that S is a surface-active element and hence the sulphur concentration at the solid/liquid interface would be much higher than in the bulk liquid^16^. A consequence of this argument is that MnS phases will precipitate at the solid/liquid interface as was experimentally found. See Figs 5(e–h) and 6(e–h).

MnS precipitation in liquid steel is more likely to occur in a high carbon steel compared to a low carbon steel because the liquidus temperature is decrease by an increase in carbon content, which reduces ${\rm{\Delta }}{G}_{MnS}^{0}$ in Eq. 4. Also, a higher carbon concentration in liquid steel increases the sulphur activity due to strong thermodynamic interaction as shown in Table 3, which encourage MnS precipitation from liquid steel. In addition, our *in situ* observations revealed that MnS precipitates on the surface even when the assumed actual product [%Mn]_pre_ × [%S]_pre_ is lower than the theoretical solubility product. This finding is new and helpful in order to avoid unexpected MnS precipitation not only at the solid/liquid interface, but also at the interface between liquid steel and gas pertaining to the widely used steel refining processes.

### Type B MnS crystallisation in solid steel

As discussed above, Type A MnS precipitates only on the solid/liquid interface due to the fact that sulphur is a surface-active agent. During solidification, the sulphur concentration at the steel surface is higher than in the bulk liquid and hence, it is easier for MnS to crystallise at the surface. However, solid steel still contains sulphur and manganese and the solubility product of MnS in the solid steel is reducesd as the temperature drops^11,12^ and hence, MnS can form in the solid. In this section, the crystallization of Type B MnS in the solid as well as on the surface is considered.

**Figure 7:**
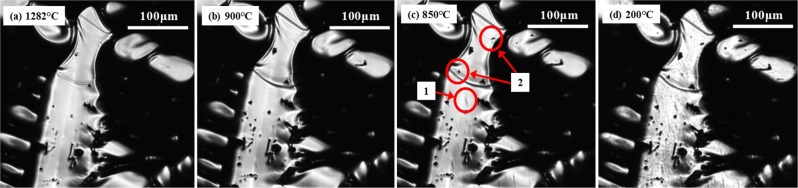
*In situ* observation of MnS crystallisation on the surface of 95 C steel cooled at a rate of −100 °C/min. ((**a**) The solidus temperature 1282 °C, (**b**) 900 °C, (**c**) 850 °C, (**d**) 200 °C).

MnS crystallisation in the solid steel was studied using the Type-2 furnace, which provides a uniform composition distribution within the sample. Figure 7 shows *in situ* observations of Type B MnS crystallisation on the surface of 95 C steel cooled at a rate of −100 °C/min, derived by CSLM. Figure 7(a) shows the steel surface at its solidus temperature 1282 °C. The black dots in image (a) are non-metallic inclusions, which existed in the liquid phase and were engulfed in the solid phase. As shown in the image (b-c), a rod-like phase in position 1 and a small particle in position 2, crystallised between 850 and 900 °C. These new phases keep their morphologies on cooling to room temperature as shown in Fig. 7(d).

**Figure 8:**
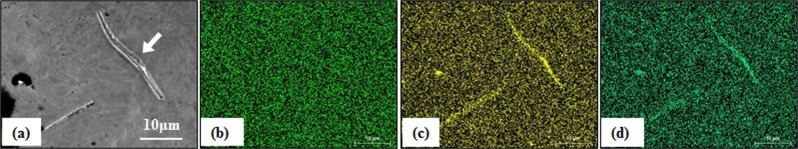
SEM (SE) image (**a**) and EDS mappings (**b**–**d**) of a rod-like MnS phase (type B-1) on the surface of 95 C steel ((**b**) oxygen, (**c**) sulphur and (**d**) manganese).

**Figure 9:**
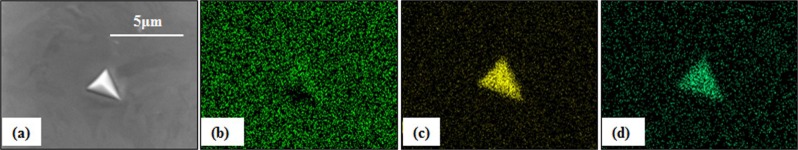
SEM (SE) image (**a**) and EDS mappings (**b**–**d**) of a polygonal MnS phase (type B-2) on the surface of 95 C steel ((**b**) oxygen, (**c**) sulphur and (**d**) manganese).

**Figure 10:**
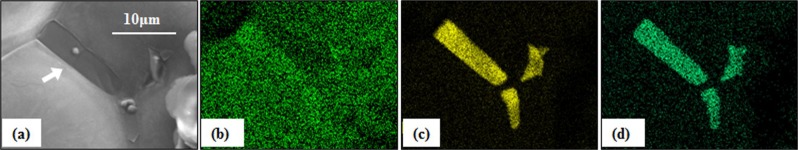
SEM (SE) image (**a**) and EDS mappings (**b**–**d**) of MnS phases at the boundary (type B-3) on the surface of 95 C steel ((**b**) oxygen, (**c**) sulphur and (**d**) manganese).

SEM-EDS analysis of a rod-like phase (Type B-1), shown as position 1 in Fig. 7(c), is shown in Fig. 8. This type of phase was identified as MnS and was observed in both steels cooled at low cooling rates, −5 and −100 °C/min. Figure 9 presents SEM-EDS analysis of another type of MnS particle Type B-2 Fig. 9 shows that this MnS particle of polygonal-shaped phase was found on the surface of both steels at cooling rates between −5 and −2000 °C/min. Another type of MnS phase (Type B-3), formed at the boundary on the surface, Fig. 10. This type of MnS is larger than Type B-1 and B-2 and is found at grain boundaries and presumably formed in the final stages of solidification. This type of MnS was identified in both steels, cooled at cooling rates of −100, −500 and −2000 °C/min. This phase was not observed in the CSLM because the area of observation in the microscope is limited and in addition, the very shallow depth of field in the laser microscope renders observation difficult in the final stages of solidification when significant shrinkage occurs.

Table 5 summarises the identification of the three types of MnS phases described above under the different experimental conditions. Type B-1 and B-2 formed at slow cooling rates (−5 and −100 °C/min). This observation is consistent with the previous study conducted by Yuki *et al*.^24^, in which rod-like and triangularly shaped MnS phases were found on the surface of an Fe-Ni alloy cooled at a rate of −20 °C/min. However, Type B-1 MnS was not found at high cooling rates of −500 and −2000 °C/min. The implication of this observation is that it takes longer for rod-like MnS precipitates to crystallise and grow than for polygonal particles. Type B-3 MnS precipitates were present at all but the lowest cooling rate of −5 °C/min. Because manganese as well as sulphur are surface-active elements, the concentration thereof is higher at grain boundaries and for this reason very large Type B-3 MnS precipitates form at grain boundaries in the solid steel at low cooling rates. When the cooling rate is very slow there is enough time for diffusion of the Mn and S from grain boundaries into the solidified grains, hence, we do not have accumulation of these elements at grain boundaries.

**Table 5:** Identification of three types of MnS phase on the surface of 60 C and 95 C steel (O: identified, X: not identified).

	Cooling rate (°C/min)	Type B-1	Type B-2	Type B-3
		Rod-like MnS	Polygonal MnS	MnS at the boundary
60 C steel	−5	O	O	X
	−100	O	O	O
	−500	X	O	O
	−2000	X	O	O
95 C steel	−5	O	O	X
	−100	O	O	O
	−500	X	O	O
	−2000	X	O	O

**Figure 11:**
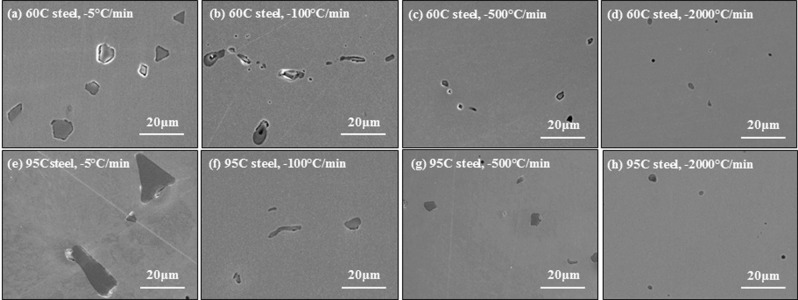
MnS phases in the cross sectional SEM (SE) images of (**a**–**d**) 60 C steel and (**e**–**f**) 95 C steel with cooling rates of (**a**,**e**) −5 °C/min, (**b**,**f**) −100 °C/min, (**c**,**g**) −500 °C/min and (**d**,**f**) −2000 °C/min.

Figure 11 presents SEM images (SE) obtained from cross sections of steels 60 C and 95 C, cooled at cooling rates between −5 and −2000 °C/min. These pictures were taken following mirror polishing with no etching. The darker phases identified in each image are MnS inclusions.

### Type C MnS at the oxide particle interface

**Figure 12:**
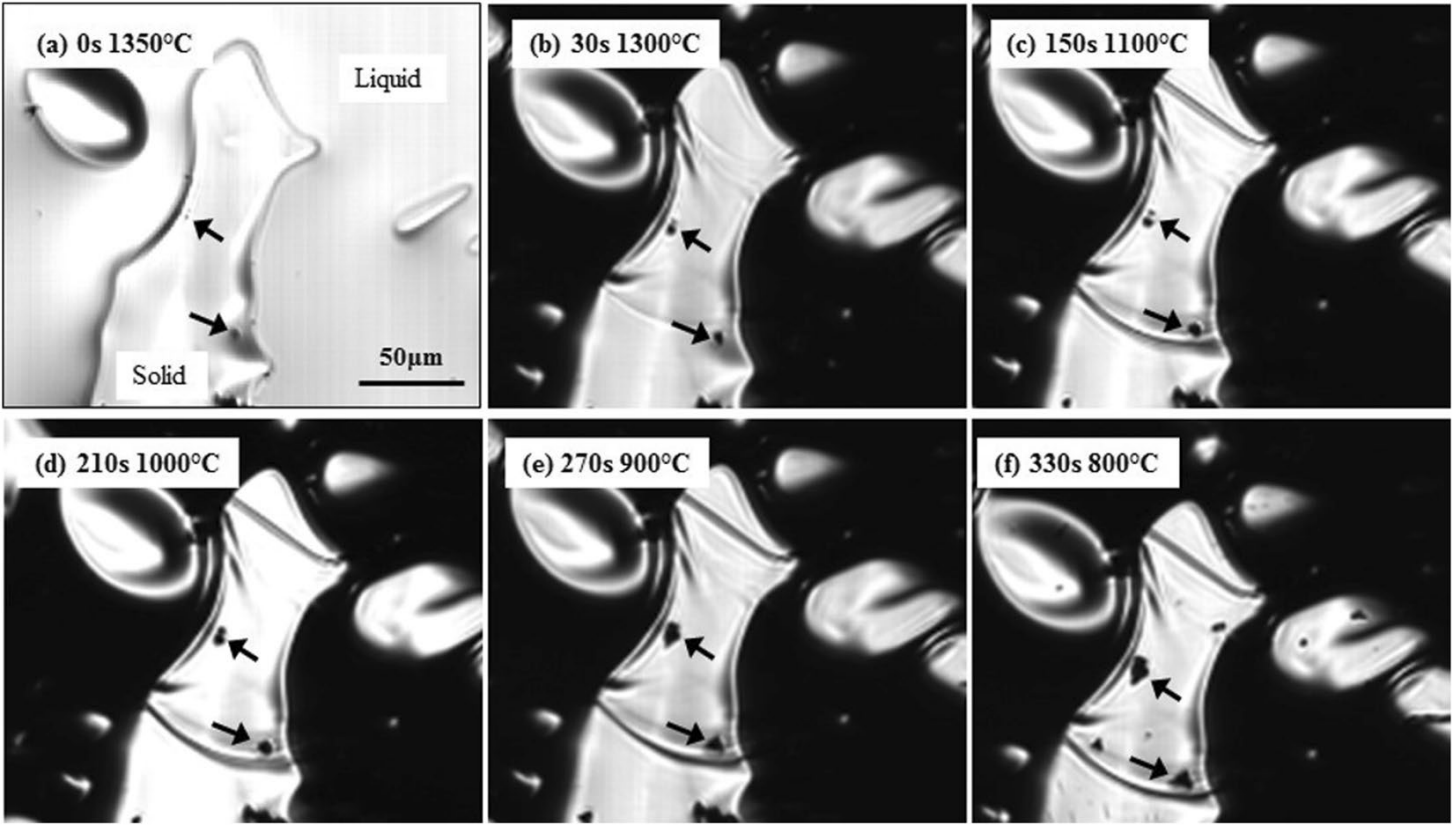
*In situ* observation of MnS formation at oxide particles in solidified 95 C steel in type-2 furnace during cooling with −100 °C/min. ((**a**) 0 s, 1364 °C, (**b**) 30 s, 1300 °C, (**c**) 150 s, 1100 °C, (**d**) 210 s, 1000 °C, (**e**) 270 s, 900 °C, (**f**) 330 s, 800 °C).

One of the nucleation site for MnS formation is oxide inclusions which will form a duplex MnS-oxide inclusion. MnS formation at the interface between oxide inclusion and steel was identified *in situ* by both Type-1 and Type-2 furnaces when the steel was cooled at cooling rates of −500 °C/min or lower. Figure 12 shows formation of this type of MnS in Steel 95 C cooled at a rate of −100 °C/min in Type-2 furnace. Firstly, the oxide particles indicated by arrows are engulfed in the solidifying shell, as shown in Fig. 12(a). Just after the engulfment, their morphologies gradually changing from small particles to irregular shaped particles and the area of the particle is expanding as temperature drops, as shown in Fig. 12(b–f). More importantly, while a single MnS starts crystallising at approximately 900 °C in the absence of oxide particles, as shown in Fig. 7, MnS is forming at the oxide particle interface with steel at higher temperature, approximately 1300 °C.

**Figure 13:**
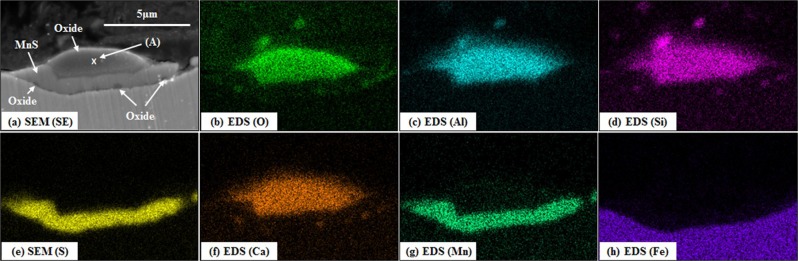
A cross-sectional SEM (SE) image (**a**) and EDS mappings (**b**–**h**) of a duplex inclusion on the surface of 95 C steel cooled with −100 °C/min. ((**b**) oxygen, (**c**) aluminium, (**d**) silicon, (**e**) sulphur, (**f**) calcium, (**g**) manganese and (**h**) iron).

**Figure 14:**
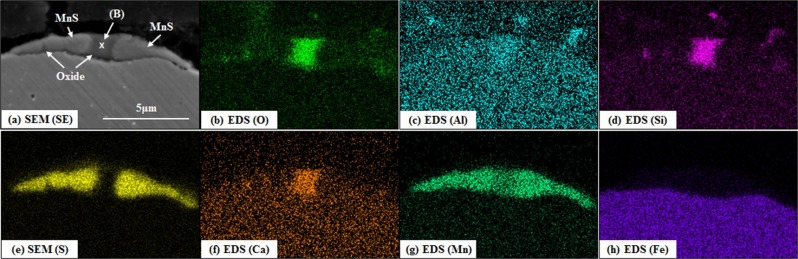
Cross-sectional SEM (SE) image and EDS mappings of a duplex inclusion on the surface of Steel 95 C cooled at a rate of −100 °C/min. ((**b**) oxygen, (**c**) aluminium, (**d**) silicon, (**e**) sulphur, (**f**) calcium, (**g**) manganese and (**h**) iron).

A cross-sectional SEM-EDS analysis of a duplex inclusion on the surface of Steel 95 C l is presented in Fig. 13. the inclusion is a mixture of MnS and oxide phases. MnS is positioned at the bottom of the oxide phases, but the small particles of other oxide phases can be also identified at the bottom part of MnS phase. It appears as if MnS grew up towards the inside the oxide phase during cooling. Liu *et al*.^25^ argued that the source of sulphur in the MnS phase is dissolved sulphur in liquid oxide. Similarly, it seems that the MnS shown in Fig. 13 formed from dissolved sulphur in the oxide phase and manganese dissolved in either the oxide phase or base steel. These changed in the composition of inclusions has been discussed in detail in other paper by authors. Another duplex inclusion is shown in Fig. 14. The duplex inclusion in this figure is a mixture of MnS and oxide phases, and MnS it seems as if the MnS grew up inside the oxide phases as in the same way as the MnS shown in Fig. 13. However, while MnS is positioned at the bottom of oxide phase in Fig. 13, MnS phase reached the top of oxide phase in Fig. 14.

The chemical compositions of the oxide phases of the duplex inclusions, indicated as position (A) in Fig. 13 and position (B) in Fig. 14, are summarised in Table 6. These data were derived by conducting SEM-EDS quantitative point analysis, and these values are normalised to 100 percent. In both positions (A) and (B), small amounts of sulphur and iron were detected due to the influence of a nearby MnS inclusion and the base steel respectively. By excluding these two elements, the oxide phase is considered to consist of Al_2_O_3_, SiO_2_, CaO and MnO.

**Table 6:** Chemical compositions of the oxide phases of the duplex inclusions calculated by SEM-EDS point analysis (unit: wt%).

	O	Al	Si	S	Ca	Mn	Fe
Position (A)	33.2	14.7	10.8	0.1	26.5	4.4	1.9
Position (B)	28.8	1.0	12.1	1.8	7.2	41.7	5.0

Liquid fractions at position (A) and (B) in the temperature range 1000 to 1400 °C were estimated by using FactSage 7.0 with Foxid databases. Al, Si and Ca detected in Table 6 are oxidized to Al_2_O_3_ and SiO_2_ and CaO. The MnO content was estimated by Eq. 10, assuming that all detected sulphur is combined with manganese, and the residual manganese exists as a form of MnO.

10 $$( \% \text{MnO})=\{( \% Mn)-( \% S)\cdot \frac{54.94}{32.07}\}\cdot \frac{70.94}{54.94}$$

**Figure 15:**
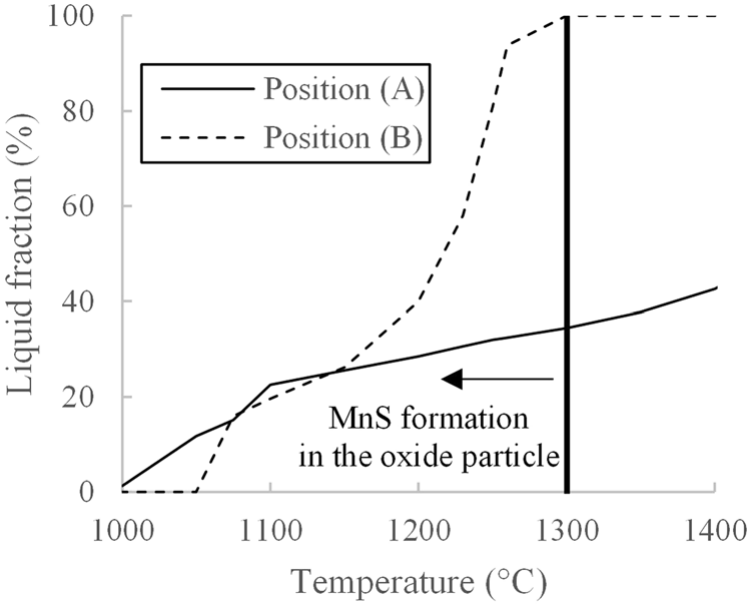
Liquid fraction transitions of oxide phases indicated in position (A,B).

As illustrated in Fig. 15, while position (A) is semi-solid phase at the beginning of MnS formation at 1300 °C, position (B) is fully-liquid phase. Additionally, the liquid fraction of (B), in which MnS growth reached the top, is higher than that of (A) until approximately 1150 °C. Although the liquid fraction was predicted using the composition data at room temperature, it is presumed that a MnS nucleus formed at the interface between the steel and the oxide phases with high liquid fraction, is capable of growing up higher into the oxide phase. If the liquid fraction is low, the MnS nucleus is less likely to grow inside the oxide phase. Instead, it is forced to spread the bottom of oxide phase, as presented in Fig. 13.

It is important to note that this phenomenon described above is completely different from the behaviour of non-metallic inclusions in Al-killed, low carbon steel. Alumina clusters and spinel (Al_2_O_3_-MgO), which are well-known inclusions in Al-killed, low carbon steel, have considerably higher melting points and they are fully solid at the temperature of the steel refining process. Most high carbon steels are deoxidized by silicon instead of aluminium in order to avoid such detrimental solid inclusions. The findings in the present study provides important evidence that MnS nuclei grows inside semi-liquid inclusions. These findings will contribute to further understanding of behaviour of inclusion in high carbon steel.

## Conclusion

In this study, different stages of formation of MnS and different types of MnS that precipitate in high carbon steels have, for the first time been investigated by re-melting and cooling at different cooling rates. The main findings are:

1. MnS precipitation on the surface of liquid steel (Type A) was observed *in situ* by utilizing a concentric solidification technique in high-temperature laser-scanning confocal microscopy. More MnS precipitated at higher cooling rates because of the built-up of soluble elements at the solid/liquid interface. MnS can precipitate in the liquid even when the solubility product of MnS does not reach the theoretical value because sulphur is a surface-active element, which increases its concentration on the solid/liquid surface.
2. Three types of single MnS phase (Type B) crystallised on the surface of solidified high-carbon steels. Rod-like MnS (Type B-1), polygonally shaped (Type B-2) and MnS on grain boundaries (Type B-3) were identified.
3. In addition to them, the single MnS phase was also crystallised in the bulk steel (type B-4), and its mean size becomes smaller under higher cooling rate.
4. MnS formation at the oxide particles on the steel surface (Type C) was observed *in situ*. Cross sectional SEM-EDS analysis revealed that MnS formed within the oxide phase. When there is a large liquid fraction of oxide at the temperature of MnS formation, MnS nuclei grew to the top of the oxide phase. Alternatively, MnS force spreads throughout the interface between the oxide and base steel.
